# Impact of *Mycobacterium ulcerans* Biofilm on Transmissibility to Ecological Niches and Buruli Ulcer Pathogenesis

**DOI:** 10.1371/journal.ppat.0030062

**Published:** 2007-05-04

**Authors:** Laurent Marsollier, Priscille Brodin, Mary Jackson, Jana Korduláková, Petra Tafelmeyer, Etienne Carbonnelle, Jacques Aubry, Geneviève Milon, Pierre Legras, Jean-Paul Saint André, Céline Leroy, Jane Cottin, Marie Laure Joly Guillou, Gilles Reysset, Stewart T Cole

**Affiliations:** 1 Unité de Génétique Moléculaire Bactérienne, Institut Pasteur, Paris, France; 2 Groupe d'Etude des Interactions Hôtes Parasites et Animalerie Hospitalo-Universitaire, Université d'Angers, Angers, France; 3 Equipe Avenir Inserm, Biology of Intracellular Pathogens, Institut Pasteur Korea, Seoul, South Korea; 4 Unité de Génétique Mycobactérienne, Insitut Pasteur, Paris, France; 5 Plate Forme 3-Protéomique, Insitut Pasteur, Paris, France; 6 Université René Descartes, Paris, France; 7 Inserm U570, Paris, France; 8 Université de Nantes, Nantes, France; 9 Inserm U601, Nantes, France; 10 Unité d'Immunophysiologie et Parasitisme Intracellulaire, Institut Pasteur, Paris, France; University of Washington, United States of America

## Abstract

The role of biofilms in the pathogenesis of mycobacterial diseases remains largely unknown. *Mycobacterium ulcerans,* the etiological agent of Buruli ulcer, a disfiguring disease in humans, adopts a biofilm-like structure in vitro and in vivo, displaying an abundant extracellular matrix (ECM) that harbors vesicles. The composition and structure of the ECM differs from that of the classical matrix found in other bacterial biofilms. More than 80 proteins are present within this extracellular compartment and appear to be involved in stress responses, respiration, and intermediary metabolism. In addition to a large amount of carbohydrates and lipids, ECM is the reservoir of the polyketide toxin mycolactone, the sole virulence factor of M. ulcerans identified to date, and purified vesicles extracted from ECM are highly cytotoxic. ECM confers to the mycobacterium increased resistance to antimicrobial agents, and enhances colonization of insect vectors and mammalian hosts. The results of this study support a model whereby biofilm changes confer selective advantages to M. ulcerans in colonizing various ecological niches successfully, with repercussions for Buruli ulcer pathogenesis.

## Author Summary

Mycobacterium ulcerans is the etiologic agent of Buruli ulcer, a necrotic skin disease affecting humans living close to wetlands in tropical countries. This mycobacteria resides in water where it could colonize many ecological niches such as aquatic plants, herbivorous animals, and water bugs. The latter were shown to be able to transmit the bacteria to mammalian hosts. Here, we described that the bacilli could be structured with a thick envelope called the extracellular matrix (ECM). This peculiar coat contains in small vesicles a toxin named mycolactone, the main virulence factor of M. ulcerans. The ECM confers to the mycobacterium increased resistance to antimicrobial agents and plays a role in virulence. Indeed, a bacteria with ECM is more potent for colonization of insect vectors and mammalian hosts compared to bacteria. Unraveling the regulation of the production of the ECM together with the export of mycolactone will be an important step in developing new pharmacological approaches for the treatment of Buruli ulcer, which has been greatly handicapped by the lack of effectiveness of the current antibiotics.

## Introduction

Mycobacterium ulcerans is the etiologic agent of Buruli ulcer, a necrotic skin disease affecting humans living close to wetlands in tropical countries. The natural history and transmission of this mycobacteria are still obscure. Epidemiological studies suggest that swampy areas, and more specifically, aquatic environments, are the main ecosystems inhabited by M. ulcerans [1–7]. Many aquatic insects in this environment are predators that feed on herbivorous organisms, such as snails, which, after grazing on plants covered by *M. ulcerans,* act as passive hosts. It is conceivable that, following ingestion of prey contaminated with *M. ulcerans,* certain carnivorous aquatic insects might then transmit the bacteria to humans. We previously demonstrated that predatory aquatic insects, such as *Naucoris cimicoides,* ingest M. ulcerans–containing prey under laboratory conditions and are both hosts and vectors of this mycobacterial species [8–11]. Indeed, they are able to deliver invasive bacteria to laboratory mice, whose tails were exposed to the infected insects, with lesions developing 30 to 90 d later precisely at the point where the bite occurred.

M. ulcerans is the only *Mycobacterium* species that localized within the salivary glands of these aquatic insects, where it can both survive and actively multiply without damaging insect tissues [8,10]. Furthermore, it has recently been shown that the lipid toxin mycolactone, the sole known virulence factor responsible for Buruli ulcer [12], is essential for the colonization of the salivary glands and that mycolactone-deficient mutants do not multiply in N. cimicoides [10].

Another striking feature of M. ulcerans is its ability to assemble into a biofilm, as first seen on the surface of aquatic plants [3]. Biofilms for human bacterial pathogens such as *Pseudomonas aeruginosa, Haemophilus influenzae,* and *Vibrio cholerae* have been well-studied [13] and consist of discrete bacteria surrounded by an extracellular matrix (ECM) [14,15]. Typically, the ECM shapes the bacterial network and can be crossed by channels, which play a critical role in water and nutrient circulation, as well as in interbacterial communication via quorum-sensing [16].

Biofilm formation confers a selective advantage for persistence under diverse environmental conditions and for resistance to antimicrobial agents, and also facilitates colonization of the host by the bacteria [13]. With regard to mycobacterial species, mutants of *M. avium* and M. smegmatis impaired in biofilm formation are less able to invade and translocate through bronchial epithelial cells and to form smegma in mice, respectively [17,18]. Molecular events involved in biofilm formation have already been reported in several studies undertaken on the genetically tractable M. smegmatis [19]. Recently, the GroEL1 chaperone was shown to be involved in mycolic acid biosynthesis during biofilm formation.

Here, we show that the ECM of M. ulcerans differs from known biofilms since it is associated with only the outermost cell layer as opposed to classic biofilms in which all cells are surrounded by the matrix. Biochemical characterization of the ECM was performed and its role in pathogenesis at the different stages of the currently known life cycle investigated. Taken together, these findings provide insight into the factors that promote persistence in diverse environmental niches and infectivity of M. ulcerans to various hosts.

## Results

### M. ulcerans Colonies Are Covered by a Novel Type of ECM Harboring Vesicles

**Figure 1:**
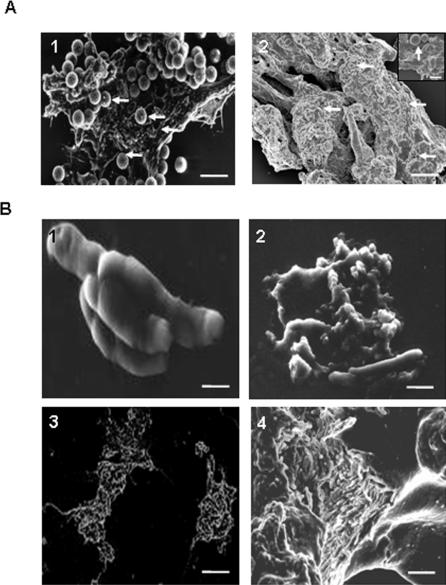
M. ulcerans ECM and Its Formation (A) Scanning electron micrographs of bacilli isolated by immunomagnetic beads separation from mammalian host tissues. Bacilli isolated from human (1) or mouse (2) tissue formed large clusters that are surrounded by ECM. Arrows indicate the immunomagnetic beads. Scale bar: 10 μm (1 and 2), 1 μm (inset). (B) Scanning electron micrographs of cluster formation by M. ulcerans. (1) Ten days after inoculation in 7H9 culture medium supplemented with OADC and containing Tween 80 0.05%, some bacilli formed a small cluster. (2) After 20 d, the bacilli had multiplied and the clusters were covered by extracellular material. (3) At the end of exponential growth (45 d after inoculation) a large cluster was formed (4), which was surrounded by the ECM. Scale bars: 1 μm (1), 1.5 μm (2), 25 μm (3), and 10 μm (4).

Given the complexity of the life cycle of *M. ulcerans,* systematic examination was undertaken of the ultrastructure of the bacterium by scanning electron microscopy. Large clusters of M. ulcerans that were covered with a biofilm-like structure were detected in biopsy samples from patients with confirmed Buruli ulcers (Figure 1A). An analogous biofilm structure was also found in bacteria isolated from lesions from mice experimentally infected with M. ulcerans. According to the relative magnetic bead size, the surface of the recovered biofilm-like structure is estimated at about 200 μm × 50 μm, suggesting that one structure could harbor up to 10^5^ bacteria. Further analysis of the same samples by Ziehl–Neelsen staining also revealed that, for each biofilm-like structure, there were less than ten free individual bacilli (unpublished data). A biofilm-like structure was also seen with M. ulcerans cultured in 7H9 broth, with or without Tween 80, and in 7H11 and 7H12 with or without PANTA (antimicrobial mixture) (unpublished data). The same amount of ECM was recovered from bacteria grown under all these culture conditions.

**Figure 2:**
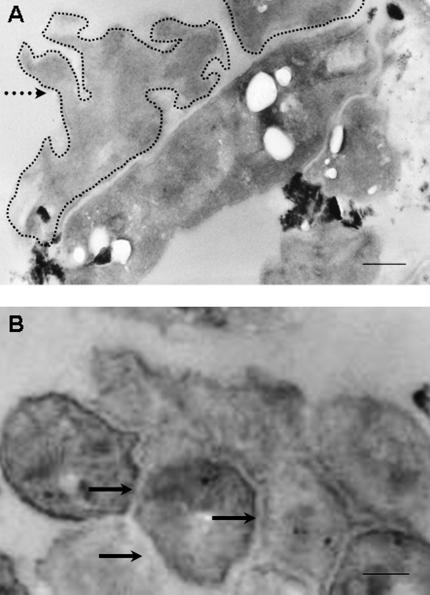
M. ulcerans ECM Localized Only in the Outermost Bacterial Layer Transmission electron micrographs of cluster formation by M. ulcerans showed that ECM covers only the peripheral bacterial layer. (A) ECM thickness is estimated to range between 4 and 40 μm (dotted line and arrow). (B) Compared to classical biofilm, the space between M. ulcerans bacteria in the cluster is small (arrows), with little ECM. Scale bars: 1 μm.

**Figure 3:**
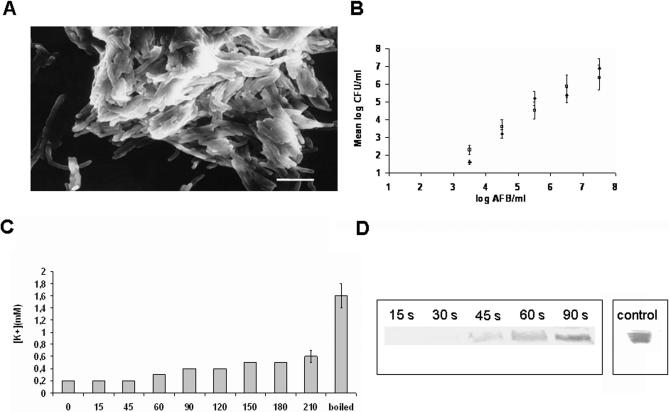
Impact of ECM Removal on M. ulcerans Cultivability and Permeability (A) Scanning electron micrographs of M. ulcerans recovered after ECM extraction. The ECM could be removed by vortexing the cells with glass beads in Tween 80 0.05%. Scale bars: 5 μm. (B) The effect of removal of ECM on the growth of M. ulcerans. CFUs onto Löwenstein–Jensen slants were determined after inoculation of serial dilutions of bacterial suspension (Acid Fast Bacilli [AFB]). Open symbols: CFUs from sample without ECM; filled symbols: bacterial clusters with ECM. Mean value ± standard error of the mean (S.E.M.) of three samples. (C) Potassium release assay in bacterial supernatant of M. ulcerans culture sample taken at different times after mechanical treatment with detergent for ECM removal. Each time point is the mean value ± S.E.M. of three independent assays. Boiled corresponds to the supernatant of boiled bacteria. Significant difference was obtained after 60 s of treatment. (D) Detection of KatG by Western blotting on M. ulcerans culture sample after mechanical treatment with detergent for ECM removal. Bacterial supernatant (30 μl) was taken at different times after treatment by beads and detergent was loaded on a 4%–12% SDS-PAGE gel and transferred to nitrocellulose. Detection was performed with rabbit polyclonal serum anti-KatG serum and anti-rabbit horseradish peroxidise-conjugated IgG. KatG was detected after only 45 s of treatment. Proteins (60 μg) extracted from M. ulcerans lysate were used for positive control.

**Figure 4:**
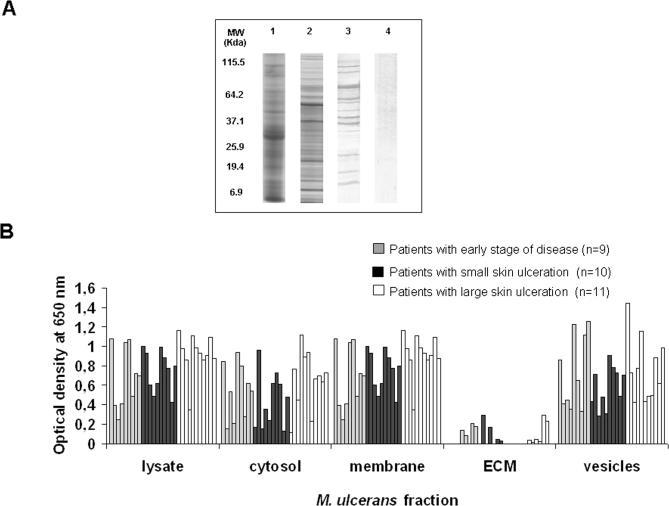
ECM Is a Poor Source of Antigens (A) Western blotting with one representative human serum sample on M. ulcerans lysate or on ECM fraction. Proteins (20 μg) extracted from M. ulcerans lysate (lanes 1 and 3) or from ECM (lanes 2 and 4) were loaded on a 4%–12% SDS-PAGE gel and transferred to nitrocellulose. Lanes 1 and 2: Coomassie staining. Lanes 3 and 4: Detection with serum from a Buruli ulcer patient, and anti-human horse radish peroxidase-conjugated IgG. Many antigens were detected in the M. ulcerans lysate, whereas none were found in ECM. (B) Detection of reactive IgG by ELISA in M. ulcerans lysate, membrane, cytosolic, ECM, and vesicle fractions. Samples were divided into three groups according to the patients' clinical stage of disease: no ulceration, limited ulceration, and extensive ulceration. Comparison using one way analysis of variance followed by the Newman–Keuls multiple comparison test shows that the relative titre of IgG binding to antigens from ECM was significantly less compared to that of the other fractions.

Strains 1615 and 1G897 were examined at different time points, and bacilli-containing clusters were evident as early as day 10 (Figure 1B-1). From days 35 to 45, large cell aggregates measuring more than 100 μm were detected (Figure 1B-3). Higher-magnification micrographs revealed that entire clusters were surrounded by an abundant biofilm-like structure, the ECM (Figure 1B-4). All M. ulcerans clinical isolates that were cultured under the same conditions displayed the same biofilm-like structure, including the mycolactone-deficient mutant of 1615, *mup045* (unpublished data). In contrast, other environmental or pathogenic mycobacteria, such as *M. chelonei, M. fortuitum, M. kansasii,* and *M. tuberculosis,* grew in vitro without displaying significant ECM despite their exhibiting large clusters of cells (Figure S1). *M. marinum,* the progenitor of M. ulcerans [20], formed discrete packets of cells that were quite distinct from the clusters, but displayed no ECM (Figure S1-3). These data show that the ECM is a peculiar feature of M. ulcerans colonies.

Subsequent analysis by transmission electron microscopy (TEM) revealed that the ECM covers only the outermost bacterial layer, its thickness was estimated to range between 4 and 40 μm (Figure 2A, dotted circled area). Strikingly, very little matrix was found within the bacterial network (Figure 2B, arrows). This contrasts with classical biofilm, in which the bacteria are each individually surrounded by the matrix [14,15].

Furthermore, scanning microscopic pictures of M. ulcerans strains revealed the presence of vesicles on the surface of the ECM after 35 to 45 d of incubation (Figure 3A). The diameter of the vesicles varied between 50 and 200 nm (Figure 3B). Vesicles were isolated by ultracentrifugation from the wild-type bacterial ECM as well as from the mycolactone-deficient mutant (unpublished data). Together with ECM, vesicles could also be recovered from biopsies of mouse lesions by immunomagnetic separation (Figure 3C). Furthermore, the vesicles could be isolated independently from the ECM fraction by performing ultracentrifugation and were thus considered separately in the following analysis.

To determine whether this matrix influences bacterial phenotype, comparison of the growth rate of M. ulcerans either harboring ECM, or from which ECM was carefully removed, was undertaken. No difference in colony-forming unit (CFU) counts of bacteria using Löwenstein–Jensen slants, and in metabolic activity using the Bactec radiometric method, was found with or without ECM removal from M. ulcerans (Figure 4), suggesting that biofilm does not confer a selective advantage for bacterial growth in vitro. In our culture conditions, the ECM re-forms in 2 wk.

### ECM Isolation and Effect of Its Removal on M. ulcerans Cultivability and Permeability

The ECM fraction was isolated from broth-cultured M. ulcerans by mechanical disruption combined with Tween 80 detergent treatment, as typically used for other mycobacteria [21,22]. Fifteen seconds are sufficient for complete removal of ECM from bacteria (Figure 4A). We then compared the effect of the treatment on the cultivability of the treated bacteria. The same amount of CFUs was obtained for M. ulcerans with or without ECM (Figure 4B), showing that this mechanical disruption does not impair the cultivability of the bacteria. Furthermore, we checked whether this treatment modifies bacterial permeability. To this end, the level of potassium release by bacteria was monitored. No significant levels of potassium were released after mechanical vortexing for up to 60 s compared to untreated bacteria (Figure 4C). In addition, the presence of KatG, a catalase-peroxidase that is cytosolic or membrane-associated, was not detected by Western blot analysis in samples that had been treated for 15 s and 30 s (Figure 4D). Altogether, these data show that ECM can be efficiently isolated with a 15-s mechanical disruption, and this argues against major contamination of ECM by lysed bacteria.

### The ECM Protects Bacteria against Antimicrobial Agents

To determine whether ECM plays a role in protecting bacteria from toxic compounds in the environment, the susceptibility of *M. ulcerans,* with and without ECM, toward chlorine and two common antibiotics was tested. This could be done since removal of ECM did not alter the growth of *M. ulcerans,* as shown above (Figure 4B). The minimum inhibitory concentration (MIC) of rifampin was <0.0625 μg/mL for M. ulcerans devoid of ECM compared to 0.5 μg/mL for M. ulcerans with ECM, a significant increase in susceptibility (*p* = 0.01). In contrast, the MIC for amikacin was identical under both experimental conditions. To test the susceptibility of M. ulcerans to chlorine, the bacteria were incubated in solutions of different concentrations. The concentrations of chlorine required to kill 10^8^ M. ulcerans cells, with or without ECM, were 100 and 40 mg/l, respectively (*p* = 0.05). Taken together, these results suggest that the ECM protects the M. ulcerans population from noxious agents.

### The M. ulcerans ECM Contains Numerous Proteins Involved in Stress Responses and Intermediary Metabolism, but No Antigens

**Figure 5:**
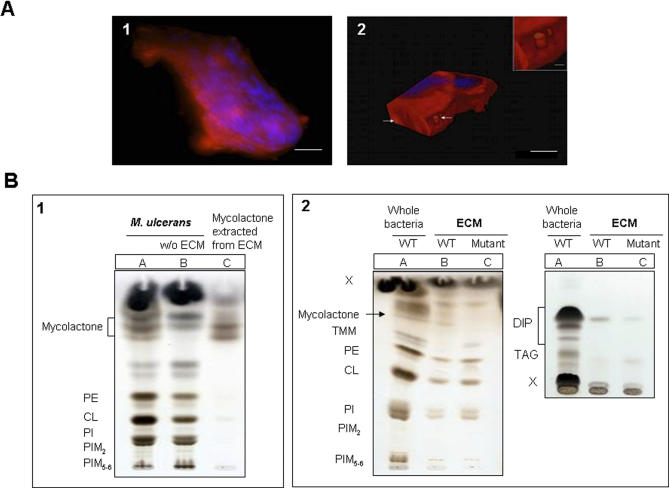
Analysis of Carbohydrate and Lipids in ECM (A) Detection of carbohydrates in M. ulcerans aggregates by multicolor fluorescence microscopy*.* (1) Bacteria were stained by DAPI, whereas carbohydrates labeled with Texas red hydrazide localized within an ECM. (2) After 3-D construction, channels were observed (arrows and insert) in ECM. Scale bars: 10 μm; insert, 1 μm. (B) TLC analysis of total lipids of M. ulcerans and purification of mycolactone from ECM. (1) The ECM of M. ulcerans wild-type strain was extracted with glass beads and Tween 80. The TLC plate was developed in CHCl_3_/CH_3_OH/H_2_0 (65:25:4, vol:vol). Lane A: Total lipids of M. ulcerans with ECM. Lane B: Total lipids of M. ulcerans without ECM. Lane C: The lipids extracted from the ECM were fractionated by adsorption chromatography on a silica gel column and mycolactone was purified by preparative TLC. (2) Lane A: Total lipids extracted from wild-type M. ulcerans before the treatment with glass beads. The ECM of M. ulcerans wild-type (WT) (lane B) and mutant (Mut) strains (lane C) was extracted with glass beads.. The TLC plate was developed in the same solvent system (left) or in petroleum ether/ethyl acetate (98:2, vol:vol, three developments) (right). Lipids were revealed with a cupric sulfate solution with charring. CL, cardiolipin; DIP, phthiodiolone diphthioceranates and phenolphthiodiolone diphthioceranates; PE, phosphatidylethanolamine; PI, phosphatidylinositol; PIMx, phosphatidylinositol mannosides (where x refers to the number of mannose residues); TAG, triacylglycerol; X, unidentified compounds.

Coomassie blue staining of an SDS-PAGE gel of the ECM fraction shows that the protein composition differs between isolated ECM and that of a whole bacterial lysate (Figure 5, lanes 1 and 2). By subsequent use of two-dimensional (2-D) gel electrophoresis or liquid chromatography combined with mass spectrometry (LC/MS), 84 proteins were identified within the ECM fraction (Table 1) and classified in seven out of 11 different functional categories used for pathogenic mycobacteria. As many as 70 (83%) of the proteins fell into four classes: 14 in virulence, detoxification, and adaptation; 10 in lipid metabolism; 32 in intermediary metabolism and respiration, and 14 in the conserved hypothetical protein class (Tables 1 and S1). AhpC, AhpD, and SodA are major players in the oxidative stress defense, whereas DnaK, GroEL1, GroEL2, GroES, HtpG, Hsp18, ClpB, ClpC1, and Clp1 are specialized in heat shock responses. Of particular interest, the GroEL1 chaperone was recently shown to modulate synthesis of mycolates during biofilm formation [19]. Strikingly, a large number of proteins essential for intermediary metabolism and respiration were identified in the ECM, which has not been reported in other bacterial biofilm studies. Among them, *pgi*-*, pgk*-*, gltA2*-*,* and *glcB*-encoded proteins are involved in glycolysis, the tricarboxylic acid cycle, or even the glyoxylate shunt.

**Table 1:**
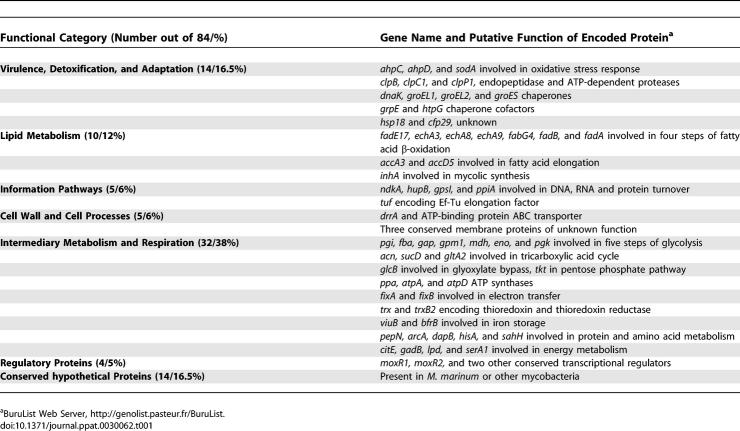
Functional Classification of Proteins Localized in the ECM of *M. ulcerans*

Furthermore, the protein pattern of isolated ECM—as classified by class or molecular weight—is significantly different from that of the secreted proteins, indicating that the ECM is an independent and specific bacterial compartment. Interestingly, besides chaperones, common mycobacterial antigens such as the antigen 85 family (FbpA, B, C, D), Mpt64, and Wag31 were not identified in the ECM by the proteomic analysis. However, it is well known that Buruli ulcer patients develop humoral responses to several M. ulcerans antigens [23,24]. To further check for the presence of M. ulcerans antigens within the ECM, this fraction was probed with the serum of 30 patients diagnosed with Buruli ulcer. Using Western blotting, no ECM-reactive IgG antibodies were detected in the serum of all patients, irrespective of the disease stage (Figure 5A). In contrast, the serum contained IgG antibodies that bound to many components of the bacterial lysate (Figure 5A). A more sensitive ELISA method was used to search for IgG antibodies recognizing M. ulcerans proteins in different fractions. Sera from all patients reacted with the whole bacterial lysate, cytosolic, membrane, and vesicle fractions, but not with the ECM fractions (Figure 5B). Indeed, no ECM-reactive IgG antibodies were detected in 53% (16/30) of the cases.

### Lipid and Carbohydrate Analysis Reveals Accumulation of Mycolactone in ECM

**Figure 6:**
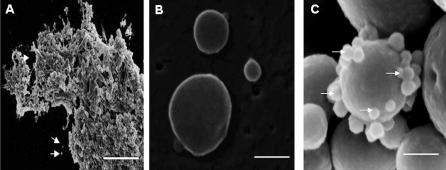
Detection and Purification of Vesicles from ECM (A) Scanning electron micrographs of *M. ulcerans* cluster showed that the ECM contains vesicles (arrows). Scale bar: 10 μm. (B) Scanning electron micrographs of vesicles purified from a culture supernatant by ultracentrifugation. The vesicle size (50 to 250 nm) is heterogeneous. Scale bar: 200 nm. (C) Scanning electron micrographs of vesicles (arrows) that were purified by immunomagnetic particle separation from an infected mouse tissue. Scale bar: 0.8 μm.

Biochemical analysis of ECM revealed a complex mixture of carbohydrates and lipids, with the total amount of carbohydrates in the ECM estimated to be as high as 2 mg per 10^9^ bacteria, a value 3-fold higher than that measured for the same number of bacterial cells lacking ECM but submitted to the same treatment. Fluorescence microscopy confirmed the abundance of carbohydrates, which are mainly localized in the ECM of the bacterial cluster (Figure 6A) Moreover, M. ulcerans aggregates have a high affinity for calcofluor, suggesting that β-glucans were a major component of ECM (unpublished data). Thin-layer chromatography (TLC) analysis of the sugar constituents of the ECM showed that glucose and mannose were the main monosaccharides found in this fraction (unpublished data).

Strikingly, the ECM of M. ulcerans wild-type strain 1615 contained the bulk of the mycolactone, with as much as 0.2 mg per gram of bacteria (Figure 6B-1). In addition to the toxin, the lipid content of the ECM from M. ulcerans wild-type strain 1615 consisted mainly of phosphatidylinositol mannosides (PIM_2_, PIM_5_, PIM_6_), phospholipids (phosphatidylethanolamine, phosphatidylinositol, cardiolipin), triacylglycerol, phthiodiolone diphthioceranates, and two unidentified apolar compounds (Figure 6B-2). Using the CS-35 monoclonal antibody, lipoarabinomannan was detected in ECM. Interestingly, in spite of their reported abundance in the outermost layers of the cell envelope of some M. ulcerans strains [25], no trehalose dimycolates were detected in the matrix. Further comparative analysis show that the type and quantity of lipids of the ECM were similar in the transposon mutant *mup045,* where they were impaired in mycolactone production, and the wild-type strain.

### Vesicles Potentiate Mycolactone Cytotoxicity

The vesicles could be isolated independently of the ECM fraction by performing ultracentrifugation. By subsequent use of LC/MS, 57 proteins were identified within the purified vesicle fraction (Table 2). Among them, only six proteins were also found in ECM, whereas 51 were also present in the membrane fraction, suggesting that vesicles are more likely to derive from the membrane compartment. Strikingly, the polyketide synthases MlsA1 and MlsB required for mycolactone synthesis are also present in the vesicles, and further lipid analysis showed the presence of mycolactone there (unpublished data).

**Table 2:**
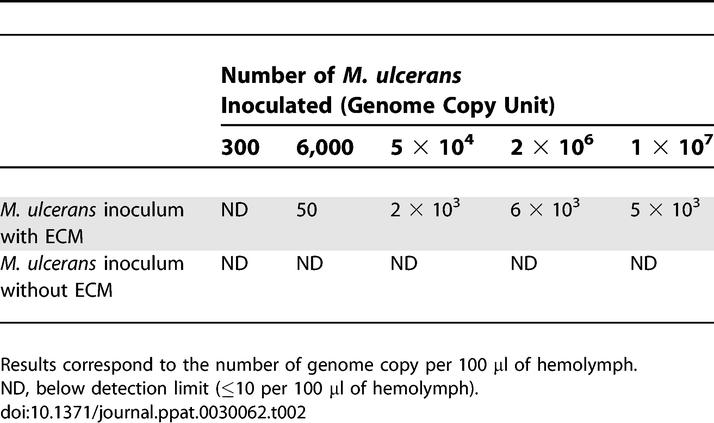
Detection of M. ulcerans DNA Isolated from *Naucoris* Insect after Infection by Grub Containing M. ulcerans with or without ECM

**Figure 7:**
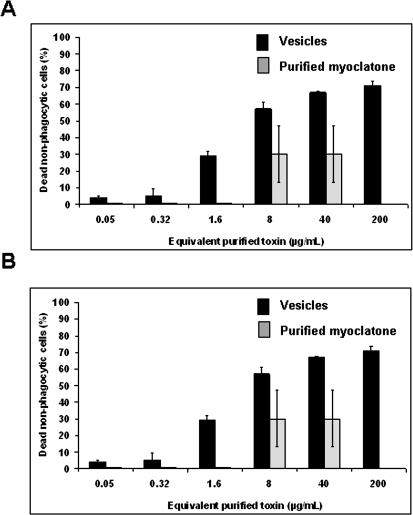
Cytotoxicity of Vesicles Comparative cytoxicity of vesicles (black bars) and purified mycolactone (grey bars) on phagocytic cells (bone marrow–derived mouse macrophages, [A]) and non-phagocytic cells (Cos cells, [B]). The mycolactone associated with vesicles is more cytotoxic than purified mycolactone.

Surprisingly, vesicles recovered from wild-type bacteria displayed cytotoxic activity on bone marrow–derived mouse macrophage cultures. Indeed, 24 h after vesicle addition, 80% of the macrophages were lysed, and similar cytotoxicity was seen with HeLa and Cos cells (Figure 7). In parallel, mycolactone purified from M. ulcerans was noticeably much less cytotoxic than the vesicles that were prepared and purified from an equal amount of the same culture, suggesting that the supramolecular organization of mycolactone affects its biological activity (Figure 7). It has also been found that, owing to its hydrophobic nature, the toxin aggregates, thus giving non-linear dose-response curves [26]. In addition, vesicles isolated from *mup045* did not cause cytotoxicity, regardless of the amount added (unpublished data), demonstrating that the vesicles' toxicity is likely due to the presence of mycolactone. Altogether, these data suggest that vesicles trigger mycolactone export, thus enhancing toxicity.

### The Presence of ECM Enhances Mouse Host Infection

To determine whether the ECM affects bacterial virulence within the host, we infected mice in the tail with *M. ulcerans,* with or without ECM, and examined the inoculation site for bacterial load, the time of onset of clinical symptoms, and symptom severity. The clinical evolution was significantly different (*p* = 0.008) using the two bacterial populations: lesions occurred much earlier in mice inoculated with 10^3^ bacilli covered by ECM (35 ± 5 d versus 58 ± 7 d). In addition, all mice (10/10) inoculated with bacteria covered with ECM showed cutaneous lesions. In contrast, seven of ten mice inoculated with bacteria lacking ECM displayed similar lesions. The results are consistent with a role for the ECM containing vesicles as a reservoir of toxin.

### ECM Is Required for Colonization of the Aquatic Insect

We investigated whether the ECM plays a role in the colonization of aquatic insects by *M. ulcerans,* as we have recently shown that the early trafficking events involve translocation of the bacillus from the head capsule to the coelomic cavity containing hemolymph [11]. To evaluate the role of ECM during the translocation, *Naucoris* aquatic insects were first fed with prey that had been inoculated with *M. ulcerans,* with or without ECM. After 6 h, the insect hemolymph was extracted and presence of M. ulcerans DNA was determined by real-time PCR. As shown in Table 2, no M. ulcerans DNA could be detected in samples from insects infected with M. ulcerans lacking ECM, whatever the initial bacterial load. In contrast, in the case of bacteria with ECM, M. ulcerans DNA corresponding to up to 5 × 10^4^ bacteria was readily detected. It should be recalled that both types of bacteria inoculated into the prey remain cultivable in this setting. Thus, in our experimental model of aquatic bug infection, M. ulcerans lacking ECM was apparently unable to colonize the insect vector, suggesting that the ECM is required during translocation.

### M. ulcerans ECM Synthesis Is Tightly Regulated

**Figure 8:**
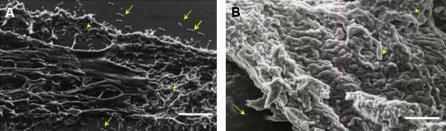
No M. ulcerans ECM Is Detected in Culture Supplemented with Organic Extracts of Algae or in Salivary Glands of Water Bugs (A) Scanning electron micrograph of the accessory salivary glands of N. cimicoides showed that the bacilli (wild-type strain) are either alone (yellow arrows) or in clusters (yellow arrowheads) lacking ECM. Scale bar: 10 μm. (B) *M. ulcerans* aggregate cultured with organic matter extracted from Rhizoclonium sp. The bacilli form large clusters that are not covered by ECM and that adhere to aquatic plant surface (arrow). Scale bar: 20 μm.

Using scanning electron microscopy, no sign of ECM was found on bacilli in the salivary glands of N. cimicoides that were previously infected with M. ulcerans covered by ECM (Figure 8A). However, the latter was observed on insect setae, through which the infection occurs via penetration of the prey [10]. Furthermore, incubation of M. ulcerans having ECM with salivary gland extracts from N. cimicoides resulted in rapid and complete degradation of ECM [11]. A likely explanation is that hydrolytic enzymes interact with the ECM and trigger its disintegration.

We have previously shown that M. ulcerans can be naturally recovered from the environment as a biofilm on the surface of aquatic algae [3]. However, this biofilm structure completely differs from that of the ECM as seen in in vitro culture in the absence of algal extract and host lesions (Figure 8B). In the presence of algal extracts, ECM could still be present, though in amounts undetectable by electron microscopy. No proteins from the ECM fraction isolated from cultures performed in the presence of algal extracts were detected by LC/MS analysis. Additionally, biochemical composition analysis showed that while significant amounts of mycolactone and (glyco)phospholipids (cardiolipin, phosphatidylethanolamine, phosphatidylinositol, and phosphatidylinositol mannosides) were detected in the ECM from control bacteria, there was no evidence for the presence of such material in algae-treated bacteria (unpublished data). Moreover, when these bacterial clusters were inoculated in fresh standard culture media, or when intermediate hosts, such as aquatic snails, were fed with these bacterial clusters, the ECM was synthesized within a few days (unpublished data).

Taken together, the absence of ECM in specific conditions, i.e., in culture medium complemented by crude algal extract or in the salivary gland of water bugs, strongly suggests regulation of ECM formation by external factors.

## Discussion

We investigated the presence of the extracellular matrix within M. ulcerans biofilms in the different settings of the currently known lifecycle. We show that M. ulcerans forms biofilms on the surface of aquatic plants [3], and, in addition, this is surrounded by an ECM in hosts such as snails, mice, and humans. We also demonstrated that M. ulcerans with ECM is more potent for insect and mammalian host colonization, which is typical for bacterial biofilms in general [13]. In contrast to other bacterial biofilms [14,15], M. ulcerans ECM is devoid of bacteria and is a thick layer only in contact with the outermost layer of M. ulcerans. While scanning electron and fluorescence microscopy unambiguously showed the abundance of ECM on the external surface of the aggregates, no ECM could detected within the network of bacteria in aggregate. This observation could not be supported by a statistical analysis, as we encountered severe difficulties for generating a large number of sections for TEM analysis from the same sample. We observed that during the fixation step, the araldite resin penetrated inefficiently into the bacterial aggregates, even when high pressure fixation was used. This phenomenon is likely to be due to the thickness of the ECM, which may also create a microaerophilic atmosphere that has been found more suitable for M. ulcerans growth in hostile conditions [27]. Similar to other biofilms, the ECM of M. ulcerans seems to be crossed by channels, whose role requires further investigation [16].

Some of the lipids/lipoglycans identified in the M. ulcerans ECM, such as lipoarabinomannan, PIM, and phthiocerol diesters, are known to play important roles in the permeability barrier of the cell envelope, as well as in the virulence of other mycobacterial species [28–30]. The ECM also contains significant quantities of carbohydrates, with glucose being the main monosaccharide constituent. The abundance of this polysaccharide in M. ulcerans suggests that it might be structurally related to the capsular D-glucan of M. tuberculosis [31]. Assuming that the bacteria use the ECM as a source of carbon and energy, there may be a relationship between the presence of carbohydrates and enzymes involved in glycolysis and the tricarboxylic acid cycle in the ECM. It has already been shown that ectoenzymes or exoenzymes of biofilms could be involved in the complex process of conversion of non-assimilable into assimilable molecules, as seen in Cellulomonas flavigena [32].

One striking feature of the ECM is its large variety and abundance of proteins. It could be argued that the presence of such a large amount of proteins results from bacterial lysis. However, the method used for preparing the ECM has also been used to investigate the architecture of the cell envelope of other mycobacteria without causing significant lysis [21], as confirmed by our control experiments (Figure 4C and 4D). Among the abundant proteins in the ECM were several chaperones, and the genes encoding chaperones like DnaK, GroEL, GroES and other oxidative stress response proteins are known to be over-expressed in different biofilms *(Staphylococcus aureus, P. aeruginosa)* [33,34], and GroEL1 was recently reported to be involved in biofilm formation in M. smegmatis [19]. Surprisingly, many enzymes required for the biosynthesis or catabolism of lipids and sugars were recovered from the ECM, whereas most of them are usually located exclusively in the cytoplasm of planktonic bacteria. Their presence within the M. ulcerans ECM suggests that they may participate in the formation or maturation of the ECM, although their functionality remains to be addressed.

Another general feature of the biofilm matrix of bacteria is its role as a diffusional barrier interfering either with the transport kinetics or the modification of extracellular molecules. We showed that ECM-coated M. ulcerans bacteria are resistant to rifampin, but not to amikacin. Similarly, biofilm-grown cells of M. avium in catheters are also resistant to rifampin as well as to clarithromycin [35]. In contrast, rifampin activity was not reduced for slime producing S. epidermidis [36]. The difference in susceptibility of ECM-harboring M. ulcerans to amikacin and rifampin could be explained by ECM acting as a selective barrier or trap for rifampin, or it may even contain enzymes that hydrolyze rifampin.

The ECM is likely to function as a resistance barrier to the host immune system. In *Staphylococcus* biofilms, the matrix was shown to interfere with macrophage phagocytic activity [37] and to prevent antibodies from reaching the bacterial cell surface [38]. On the one hand, M. ulcerans escaping from immune recognition could be due to the inaccessibility of the surface antigens to the host immune system. On the other hand, mycolactone has already been shown to limit phagocytic activity [26,39] as well as to cause the death of macrophages and other cells via apoptosis [12,40]. Uncoupling the specific effects of ECM from those of mycolactone at the cellular level is currently being investigated.

Usually, toxins of Gram-positive bacteria accumulate in the cytoplasm in a precursor form or are secreted without accumulation. In Gram-negative bacteria, apart from the endotoxin lipopolysaccharide, the same processes are observed. The presence of mycolactone in an external reservoir was proposed previously when the toxin was found associated with suspended lipids [41]. We provide further evidence by showing that mycolactone accumulates mainly in the ECM, which may play the role of a reservoir and amplify the pathogenicity of the bacteria.

Furthermore, the toxin is secreted in specialized vesicles, which are cytotoxic for a variety of cells, both phagocytic and non-phagocytic, suggesting that mycolactone-containing vesicles do not recognize a specific receptor. Similarly, vesicles have already been shown to be secreted by some bacteria and exported to reach their target for delivering virulence factors to host cells [42]. The fact that mycolactone is sequestered within vesicles suggests that treatment prospects based on the design of neutralizing antibodies against this polyketide toxin would likely be inefficient.

Although we showed that ECM has an important role for insect vector colonization and M. ulcerans translocation to the coelomic cavity, no ECM is found on the bacteria in this particular compartment. This could be due to two different processes: either hydrolysis by salivary enzymes, or down-regulation of matrix production by external factors. For instance, no matrix was produced when M. ulcerans catabolized carbohydrates present in algal material, whereas growth in lipid-rich medium such as 7H9 with oleic acid induced copious amounts. Generating a mutant lacking ECM will help decipher the molecular mechanisms involved in ECM production, although inhibiting its synthesis with plant extracts may be a useful alternative. Unraveling the regulation of the production of the ECM together with the export of mycolactone will be an important step in developing new pharmacological approaches for the treatment of Buruli ulcer, which has been greatly handicapped by the lack of effectiveness of the current antibiotics.

## Materials and Methods

### Bacterial strains.

The strains of M. ulcerans 1G897 [43] and 1615 [12] (Trudeau Collection Strain) were originally isolated from human skin biopsies from French Guyana and Malaysia, respectively. Each clinical isolate was inoculated in the mouse tail. Forty days later, M. ulcerans bacteria were recovered from the infected tissue and seeded onto Löwenstein–Jensen slants (Bio-Rad, http://www.bio-rad.com) for an additional 45 d before being aliquoted and stored at −80 °C [44]. The *mup045* mutant (MU1615::Tn*118*) has a transposon insertion located in the ketosynthase gene *mup045,* leading to undetectable levels of mycolactone in culture [26,39] The mycobacterial strains *M. chelonei* (6B0139), M. fortuitum (10B0345), M. kansasii (11B0014), M. marinum (8B0432, clinical isolate from a French patient), and M. tuberculosis H37Rv were used as controls.

### Culture conditions for M. ulcerans biofilm isolation.

Frozen aliquots of each strain were first inoculated onto 7H11 solid medium supplemented with 10% OADC (oleic acid, dextrose, catalase; Difco, Becton-Dickinson, http://www.bd.com) and Tween 80 0.05% (Sigma, http://www.sigmaaldrich.com). Then, 35 d later, exponentially growing bacteria from agar plates were harvested in 7H9 broth supplemented with 10% OADC and Tween 80 0.05% (Sigma) at 10^5^ bacteria/mL. Titration of the intitial inoculum was performed by the Shepard and Rae method [45]. The cultures were performed in 200 mL in a disposable polystyrene cell culture flask (EasYFlask; Nunc, http://www.nuncbrand.com) with gentle agitation (30 rpm) at 30 °C for 35 d, which corresponds to the end of exponential phase of bacterial growth. Regarding the experiment performed with the algal extract, mycobacteria were inoculated at 10^5^ bacteria/mL in 200 mL of 7H9 broth supplemented with 10% OADC, Tween 80 0.05%, and a crude extract of Rhizoclonium sp. algae obtained as previously described [3].

### Preparation for scanning electron microscopy.

Samples were fixed for 30 min in 0.1 M cacodylate buffer (pH 7.2) containing 2.5% glutaraldehyde for 1 h at 4 °C, then left to stand for 12 h at 20 °C in cacodylate buffer. Specimens were progressively dehydrated and then metallized prior to examination by scanning electron microscopy on a JEOL 6301F field emission microscope. For transmission microscopy, the bacteria were embedded in Araldite (Fluka, St. Quentin Fallavier, France; http://www.sigmaaldrich.com/Brands/Fluka___Riedel_Home.html). After dehydration, thin sections were stained with uranyl acetate and Reynold's lead citrate and then examined on a JEOL 120 EX electron microscope.

### Preparation for transmission electron microscopy.

The fresh broth was incubated overnight and cells were harvested by centrifugation (8,000*g* for 10 min), and washed twice in PBS. The resulting pellets were fixed in 2.5% (w/v) glutaraldehyde, in cacodylate buffer for 2 h in the dark at room temperature. Cells were washed three times in cacodylate buffer (0.1 M [pH 6.8]), postfixed for 2 h in the dark in 1% (w/v) osmium tetroxide (Sigma), 0.05% and then washed twice each in cacodylate buffer and in water. Bacteria were dehydrated through a graded ethanol series of 50%, 60%, 70%, 80%, and 95% for 5 min each, and then washed twice for 15 min each in 100% ethanol, then twice for 15 min each in propylene oxide. The bacteria were finally embedded in Araldite (Fluka). Resin was replenished the next morning and samples were left to cure at 60 °C overnight. Blocks were thin-sectioned on a Reichert–Jung microtome and mounted on copper grids. Sections were poststained with uranyl acetate and Reynold's lead citrate. Microscopy was performed on a JEOL 120 EX electron microscope.

### Human and mouse biopsy analysis.

A biopsy from an Ivory Coast patient and from mouse tail lesions were surgically excised from skin. The tissue specimens were minced with disposable scalpels in a Petri dish and ground with a Potter–Elvehjem homogenizer, size 22 (Kimble/Kontes, http://www.kimble-kontes.com), in PBS/Tween 80 0.05%. For isolation of *M. ulcerans,* the human and mouse tissue specimens were processed by immunomagnetic separation to isolate bacilli. Two types of immunomagnetic particles were used: 2.8-μm-diameter immunomagnetic particles precoated with sheep anti-rabbit IgG (Dynald) for the human sample, and 1-μm-diameter immunomagnetic particles precoated with goat anti-rabbit IgG (Interchim, http://www.interchim.com) for the mouse sample. Firstly, coating of the immunomagnetic particles (10^4^) was carried out for 2 h at 37 °C with agitation with a rabbit polyclonal antibody raised against whole PFA-fixed M. ulcerans [8] at 20 μg in a total volume 200 μl of PBS (pH 7.2) containing Tween 80 0.05%. Secondly, 0.1 g/mL of tissue homogenate was added to the coated immunomagnetic particles and incubated with bidirectional mixing at 4 °C for 12 h. Finally, particles were washed six times for 3 min each with PBS containing Tween 80 0.05%.

### ECM, vesicles, membrane, cytosolic, and secreted proteins preparation from M. ulcerans cultures.

A 35-d-old M. ulcerans shaking culture (200 mL) was washed three times with 20 mM Tris-HCl (pH 7.5) (3,000*g* for 30 min at 4 °C). The mycobacterial pellet was then resuspended in the same buffer supplemented with antiprotease Complete EDTA free cocktail (Roche, http://www.roche.com) to obtain 10^9^ bacilli/mL. Thirty glass beads (4 mm in diameter) were added to the suspension and vortexed for 15 s. The mycobacterial suspension was then centrifuged at 8,000*g* for 10 min at 4 °C. The ECM fraction consists of the supernatant collected after centrifugation and filtration through a 0.45-μm filter. The supernatant was further fractionated into vesicles that were recovered in the pellet after ultracentrifugation at 40,000*g* for 3 h, then washed three times in 0.1 M Tris-HCl (pH 7.5) with Tween 80 0.05%.

The pellet containing whole bacteria was then suspended in the same buffer and the mycobacteria were broken with 106-μm acid washed glass beads (Sigma) for 5 min at speed 30 using a bead beater (Mixer Mill MM301; Retsch GmbH, http://www.retsch.com) at 4 °C. The bacterial lysate consisted of the supernatant obtained after removal of unbroken cells and cell debris by centrifugation at 8,000*g* at 4 °C. The soluble cytosolic proteins were subsequently obtained by ultracentrifugation of the bacterial lysate (70,000*g,* 90 min, 4 °C). The pellet, consisting of membrane proteins, was washed with 200 μl of 50 mM Tris-HCl (pH 7.5) to remove residual cytosolic contaminants and then resuspended in 50 mM Tris-HCl (pH 7.5). For all fractions, quantification was performed by measuring protein concentration using a Bio-Rad protein assay. Regarding the secreted proteins, the shaking liquid culture was performed in the absence of albumin and prepared as previously reported [46]. Briefly, the culture filtrate was recovered after filtration through 0.22-μm-pore-size filters (Millex GP; Millipore, http://www.millipore.com), followed by concentration using a filter with a 3-kDa cutoff (Centricon; Millipore).

### Effect of ECM preparation on bacterial growth and bacterial permeability.

Bacterial suspension preparation and ECM removal were carried out exactly as described above. After recovery by centrifugation at 5,000*g* for 10 min, the number of CFU was determined by inoculating 10-fold dilutions of the bacterial suspension onto three Löwenstein–Jensen slants (Bio-Rad) incubating for 6 wk at 30 °C. In addition, the bacterial dilutions were inoculated into Bactec 12B vials (Becton-Dickinson) containing Middlebrook medium with ^14^C-labeled palmitic acid as a carbon source. Substrate consumption generates ^14^CO2 in the airspace of the sealed vial. The BACTEC TB-460 instrument detects the amount of released radioactivity and records it as a growth index (GI) on a scale from 0 to 999. The vials were incubated at 30 °C, and every 5 d the GI was recorded.

To test bacterial permeability, kinetics of vortexing with glass beads for the ECM removal step was performed at 15-s intervals for up to 3 min. After removal of unbroken cells and debris by filtration (0.45 μm), quantification of K+ in the supernatant was monitored on BM/Hitachi 917 apparatus following manufacturer protocol. As a control, boiled M. ulcerans suspension was used for maximum K+ release. In addition, KatG detection was performed by Western blot analysis, as this enzyme is known to be localized in the membrane and cytosol. To this end, supernatant of bacterial culture pelleted after treatment by glass bead (60 μg) was loaded on a sodium dodecyl sulfate (SDS)–polyacrylamide gel (4%–12%) (Bio-Rad) and the separated bands were transferred onto a 0.45-μm nitrocellulose membrane (Amersham, http://www.amersham.com). After blocking with 5% skimmed milk in PBS, the membrane was incubated with serum from rabbit anti-KatG 1:100 [47] in PBS containing Tween 80 0.05% (Sigma) for 90 min at 37 °C. After two washes with PBS containing Tween 80 0.05%, sheep anti-rabbit IgG (heavy and light chains) peroxidase-conjugated antibodies (Amersham) at 0.5 μg/ml and 0.5 μg/ml DAB (Interchim) was used for detection of the bands.

### Analysis of M. ulcerans proteins from ECM, vesicles, membrane, cytosolic, and secreted fractions.

Protein fractions were analyzed by 1- or 2-D gel electrophoresis followed by a combination of matrix-assisted laser desorption/ionization mass spectrometry peptide mass fingerprinting (MALDI-MS PMF) and liquid chromatography/electrospray ionization mass spectrometry (LC-MS/MS). The spots or bands of interest were excised from the gel and treated automatically using a Probest/P50MS robotics system (Genomics Solutions, http://www.genomicsolutions.com). The tryptic peptide mixture was then extracted by 10% formic acid, desalted using Micro C18 Zip Tips (Millipore), and eluted with 0.5 μl of alpha-cyano-4-hydroxy-cinnamic acid (Sigma). The samples were analyzed by MALDI-MS on a Voyager DE STR (PerSeptive Biosystems, http://www.appliedbiosystems.com) equipped with a nitrogen laser (337 nm). To search the M. ulcerans ORF database “BuruList” (http://genolist.pasteur.fr/BuruList), monoisotopic masses were assigned using a local copy of the MS-Fit3.2 part of the Protein Prospector package (University of California Mass Spectrometry Facility, San Francisco; http://prospector.ucsf.edu). The parameters were set as follows: no restriction on the isoelectric point of proteins, 50 ppm as the maximum mass error, and one incomplete cleavage per peptide. Eleven different samples were analyzed.

Protein digest fractions of samples were also analyzed by reverse phase LC-MS/MS. As peptides eluted off the C18 Pepmap column (LC-Packings, http://www.dionex.com), they were introduced on line into a QSTAR XL instrument (MDS-Sciex; Applied Biosystems, http://www.appliedbiosystems.com) and were analyzed using data-dependent switching between MS and MS/MS modes. The ProID (MDS-Sciex; Applied Biosystems) program was used to interpret the LC-MS/MS data by searching against BuruList [48]. The search parameters were as follows: 1) 0.2-Da mass error tolerance for both MS and MS/MS; 2) one missed cleavage of trypsin specificity was allowed. Peptide matches with significant homology (confidence score > 95) were considered as identified peptides. Proteins identified by a single peptide were validated by manual inspection of the MS/MS spectra.

### Human sera, ELISA, and Western blot analysis.

The Buruli Patients group consisted of 30 patients recruited from the Centre de Diagnostic et de Traitement de l'Ulcère de Buruli in Pobè, Benin, and were included in a sero-epidemiological study, for which written consent had been obtained [9]. Nine out of 30 patients presented early clinical signs without ulceration (four nodules, two oedema, three plaques), ten patients with limited ulceration (<6 cm), and 11 with extensive ulceration (>10 cm). Diagnosis of M. ulcerans infection was by Ziehl–Neelsen staining of material taken from swabs of the lesions or directly from the biopsy for the early form and confirmed by PCR for *M. ulcerans–*specific IS*2404* DNA [6]. The participants, who had given their written consent, were enrolled as volunteers in the study, the protocols of which were approved by the Ministry of Health in Benin. Serum was prepared from 8 ml of blood from each participant and tested for potential HCV and HIV exposure using Access HIV-1/2 automated immunoassay (MDA/98/58) and Sanofi Diagnostics Pasteur Access anti-HCV automated immunoassay (plus update on five other anti-HCV assays [MDA/96/26]).

Proteins (10 μg) from ECM, bacterial lysate, membrane, and cytosolic fractions were coated onto 96-well Nunc Maxisorb plates by incubation overnight at 4 °C in 100 μl of PBS containing Tween 80 0.05%. The coated plates were then incubated with PBS containing 5% skimmed milk at room temperature for 2 h. After three washes in PBS/Tween 80, the samples were incubated for 1 h at 37 °C with human serum diluted 1:200 in PBS/Tween 80. After three further washes, plate-bound human immunoglobulins were detected using peroxidase-conjugated goat anti-human IgG (γ chain) antibodies (Sigma) and OPD (Dako, http://www.dako.com). The diluted sera were tested in triplicate and the average absorbance at 650 nm was expressed in optical density units.

Proteins (60 μg) from lysates or ECM fractions were run in an SDS–polyacrylamide gel (4%–12%) (Bio-Rad), and the separated bands were transferred onto a 0.45-μm nitrocellulose membrane (Amersham). After blocking with 5% skimmed milk in PBS, the membrane was incubated with serum from humans diluted 1:100 in PBS containing Tween 80 0.05% for 90 min at 37 °C. After two washes with PBS containing Tween 80, anti-human IgG (γ chain) peroxidase-conjugated antibodies (Sigma) at 1:2,000 and 0.5 μg/ml DAB (Interchim) was used, respectively, to detect human IgGs bound to the different bands.

### Analysis by fluorescence microscopy of surface carbohydrates of M. ulcerans ECM.

Bacteria were fixed in 2.5% (w/v) formaldehyde in PBS buffer and surface carbohydrates labeled with Texas red hydrazide (Molecular Probes, http://probes.invitrogen.com) or with calcofluor white M2R (Sigma).

Bacteria were then stained by DAPI, and labeled carbohydrates were visualized directly using a Zeiss Axioskop 20 fluorescence microscope and the AxioVisionLE 4.2 SP1 program (http://www.zeiss.com) used to perform the 3-D reconstruction.

### Analysis of lipid and mycolactone contents of ECM.

ECM obtained by treating bacteria with Tween 80 0.05% and glass beads was extracted by phase partitioning. The aqueous layer from the H_2_O/CHCl_3_/CH_3_OH partition was concentrated, the polymers precipitated overnight at 4 °C with six volumes of cold ethanol, and the precipitates collected by centrifugation at 14,000*g* for 1 h. The lipoarabinomannan content of this fraction was analyzed by SDS-PAGE and immunoblotting using the CS-35 antibody as described [49]. The interphase derived from the partition experiment was extracted three times with water before precipitating the polymers with ethanol and submitting them to acid hydrolysis in 2 M trifluoroacetic acid for 2 h at 120 °C. The monosaccharide constituents of this fraction were then analyzed by TLC, and their migration profile was compared to that of known standards.

Total lipids from bacterial cells treated with Tween 80 0.05%, or untreated, were extracted as described [50] and analyzed by TLC on silica gel 60–precoated plates F_254_ (Merck, http://www.merck.de). Extraction of mycolactone and cytotoxicity tests was performed according to George et al. [40] either using the bacterial pellet (cells without ECM) or ECM [13]. The carbohydrate content of the ECM material was measured by a colorimetric method [51].

### Cytotoxicity assays.

Bone marrow–derived macrophages were obtained by seeding 10^5^ bone marrow cells from 8-wk-old C57BL/6 mice per well in RPMI 1640 supplemented with 10% heat-inactivated fetal calf serum and 10% L-cell-conditioned medium. Culture medium was changed at day 4 and just before adding mycolactone at day 7. HeLa cells and Cos cells (American Type Culture Collection, http://www.atcc.org) were cultured in Dulbecco's modified Eagle's medium supplemented with 10% heat-inactivated fetal calf serum. Proliferating cells were seeded in 96-well microtitration plates at a density of 10^5^ cells/well, which were further incubated for 24 h at 37 °C under 5% CO_2_ in air before each assay. Various concentrations of vesicles or mycolactone in ethanol were added (2 μl/well). The mycolactone used as reference was purified as previously reported [12]. After 24 h incubation in the above conditions, cytotoxicity was then assessed by addition of 20 μl of dimethylthiazolyl diphenyl tetrazolium bromide solution (MTT, Sigma) (7.5 mg/mL) to each well and further incubated for 4 h at 37 °C to allow the formation of formazan. Formazan crystals were then dissolved with 100 μl of 10 % SDS in 10 mM HCl. The optical density of each well was measured at 595 nm using a Multiwell plate reader. The values given are the average of two replicates and are representative of four independent experiments. The 50% inhibition concentration was determined by curve fitting.

### Quantification of M. ulcerans DNA in insect hemolymph using real-time PCR.

Adult N. cimicoides water bugs were collected and housed as described previously, then fed with grubs of *Phormia terrae novae* (Verminière de l'Ouest, http://www.verminieredelouest.fr) that had been inoculated beforehand with *M. ulcerans,* with or without ECM, in 30 μl by using a 25-gauge needle**.**

Six hours after feeding, the insect hemolymph was collected with an insulin syringe [10]. Pooled hemolymph (100 μl) was added to 100 μl of cold distilled water. The samples were washed three times by centrifugation (14,000*g* for 15 min) in distilled water and resuspended in 50 μl of 50 mM NaOH and heated at 95 °C for 15 min. Real-time PCR was performed using brilliant SybrGreen Q PCR mix (Stratagene, http://www.stratagene.com) containing Taq polymerase, 2.5 mM MgCl_2_, 100 μM (each) deoxynucleoside triphosphate and 20 pM primers. The primers were MLF (5'- CCCTTCGACGTCATCAAGAAA −3′) and MLR (5'- CCGACTGACCGATGAGCAA −3′), leading to amplification of a 63-bp region of the *mls* genes [52]. After 15 min at 95 °C, the DNA was amplified by 30 cycles of 45 s at 95 °C; 1 min at 61 °C, and 45 s of elongation at 72 °C on an MX3000P apparatus (Stratagene). The dissociation curve was performed between 55 °C and 95 °C.

### Susceptibility of M. ulcerans to antibiotics and chlorine.

The MIC of rifampin and amikacin, inhibiting >99% of the bacteria, was determined as previously described [53]. To measure chlorine susceptibility, ∼10^8^ bacteria, with or without ECM, were suspended in solutions containing a range of chlorine concentrations (20–200 mg per liter). After 60 min at 25 °C, residual chlorine was neutralized with sodium thiosulfate [54] and bacterial viability determined by inoculation onto Löwenstein–Jensen slants.

### Virulence assays in mice.

Suspensions (30 μl) containing 5 × 10^3^ bacteria, with or without ECM, were injected subcutaneously into the tail of ten female Balb/c mice (Charles River Laboratories, http://www.criver.com). Mice tails were examined weekly over 6 mo.

### Statistics.

The non-parametric Mann-Whitney *U* test was used for statistics. A *p*-value of < 0.05 was considered significant.

## Supporting Information

Figure S1 Scanning Electron Micrographs of Four Mycobacterial Species at the End of Exponential Growth in 7H9 Culture Medium (1) *M. chelonei;* (2) *M. kansasii;* (3) *M. marinum;* (4) M. tuberculosis. Scale bar: 5 μm.
(8.7 JPG)

Table S1 Detailed Annotation of Proteins Localized in Vesicles of *M. ulcerans*
(117 KB DOC)

## Abbreviations

CFU: colony-forming unit
ECM: extracellular matrix
LC/MS: liquid chromatography combined with mass spectrometry
MIC: minimum inhibitory concentration
TLC: thin-layer chromatography
