# Supplementary material for: Impact of Mycobacterium ulcerans Biofilm on Transmissibility to Ecological Niches and Buruli Ulcer Pathogenesis
Source: PLoS Pathog. 2007 May 4;3(5):e62. doi: 10.1371/journal.ppat.0030062 (PMC1864991; doi:10.1371/journal.ppat.0030062)
Supplement: Supplementary file 1 [file ppat.0030062.st001.doc]

Table 1S. Detailed annotation of proteins localized in vesicles of *M. ulcerans*

| **Functional Category** | **MW(Da)** | **pI** | **Gravy score** | **Gene** | | **Putative function** |
| --- | --- | --- | --- | --- | --- | --- |
| 0- **Virulence, Detoxification, Adaptation** | | | | | | |
| MUL_1393 | 56558.4 | 4.6037 | -0.052 | | *groEL2* | Chaperone protein. Prevents misfolding and promotes the refolding and proper assembly of unfolded polypeptides. Generated under stress conditions. |
| MUP032c | 149763 | 5.3942 | -0.048 | | *mlsB* | Type I modular polyketide synthase, composed of a loading module and seven extender modules; required for the synthesis of the mycolactone side-chain in *M. ulcerans*. |
| MUP040c | 179958 | 5.55 | -0.049 | | *mlsA1* | Type I modular polyketide synthase, composed of a loading module and eight extender modules; required for the synthesis of the mycolactone core in *M. ulcerans*. |
| **1- Lipid Metabolism** | | | | | | |
| MUL_1305 | 12486.9 | 3.7895 | -0.275 | | *acpM* | Involved in fatty acid biosynthesis (mycolic acids synthesis); involved in meromycolate extension. |
| MUL_1819 | 46812.3 | 10.3404 | -0.221 | | *lipO* | Unknown. |
| **2-Information pathways** | | | | | | |
| MUL_0073 | 9542.15 | 11.2788 | -0.675 | | *rpsR1* | This protein has been implicated in aminoacyl-transfer RNA binding. it appears to be situated at the decoding site of messenger RNA. |
| MUL_0766 | 43805.6 | 5.0393 | -0.311 | | *tuf* | Iron regulated elongation factor (Ef-Tu) |
| MUL_0790 | 23036.3 | 10.7514 | -0.332 | | *rplC* | This protein binds directly to 23s ribosomal RNA and may participate in the formation of the peptidyltransferase center of the ribosome. |
| MUL_0791 | 23859.9 | 10.7811 | -0.438 | | *rplD* | This protein binds directly and specifically to 23s RNA. |
| MUL_0796 | 30209.1 | 10.7105 | -0.623 | | *rpsC* | This protein is involved in the binding of initiator met-tRNA. |
| MUL_0797 | 15706.2 | 11.7371 | -0.580 | | *rplP* | This protein binds directly to 23s rRNA and is located at the site of the peptidyltransferase center. |
| MUL_0865 | 16906.5 | 10.5326 | -0.407 | | *rpsI* | Involved in translation mechanism. this protein is one of the assembly proteins of the 50s ribosomal subunit. |
| MUL_1629 | 14461.5 | 11.5523 | -0.643 | | *rplT* | This protein binds directly to 23s ribosomal RNA and is necessary to the in vitro assembly process of the 50s ribosomal subunit; it is not involved in the protein synthesizing functions of that subunit. |
| MUL_1970 | 23646 | 12.4902 | -0.580 | | *hupB* | DNA-binding protein HU homolog prevent DNA denaturation under extreme environmental conditions. |
| MUL_0793 | 30358.4 | 11.82 | -0.822 | | *rplB* | this protein is a primary 23s RNA-binding protein. it has peptidyltransferase activity. |
| **3- Cell wall and cell processes** | | | | | | |
| MUL_2616 | 77869.2 | 6.148 | 0.175 | | *ctpC* | Metal cation-transporting atpase. |
| MUL_0125 | 32249.7 | 10.3534 | -0.566 | | */* | Unknown. |
| MUL_0202 | 26283.3 | 4.7379 | -0.022 | | *pra* | Unknown. |
| MUL_0758 | 15257.6 | 6.558 | 0.273 | | *mmpS5* | Unknown, contains cog1138: cytochrome C biogenesis factor. |
| MUL_0911 | 53093.9 | 8.2248 | 0.79 | | */* | Thought to be involved in a transport system across the membrane. |
| MUL_1068 | 23832.1 | 4.3439 | -0.517 | | */* | Unknown. |
| MUL_1126 | 45679.8 | 4.4747 | -0.382 | | */* | Unknown. |
| MUL_1342 | 26846.4 | 12.0326 | -0.047 | | */* | Unknown. |
| MUL_1373 | 14240.3 | 11.8694 | 0.086 | | */* | Unknown. |
| MUL_1725 | 64287.1 | 9.2342 | -0.006 | | *secD* | Involved in protein export. part of the prokaryotic protein translocation apparatus. |
| MUL_2323 | 26862.4 | 4.8558 | -0.746 | | */* | Unknown. |
| MUL_2368 | 36544.2 | 6.1229 | 0.09 | | *lppL* | Unknown. |
| MUL_2505 | 108189 | 9.3225 | 0.031 | | */* | Unknown. |
| MUL_2842 | 29463.7 | 4.5505 | -0.481 | | */* | Unknown. |
| MUL_2842 | 29463.7 | 4.5505 | -0.481 | | */* | Unknown. |
| MUL_3059 | 71332 | 8.7618 | 0.133 | | */* | Thought to be involved in active transport of drugs across the membrane (export): multidrugs resistance by an export mechanism. responsible for energy coupling to the transport system and for the translocation of the substrate across the membrane. |
| MUL_3093 | 54118.5 | 6.412 | -0.12 | | */* | Unknown. |
| MUL_3189 | 40213.4 | 4.6626 | -0.173 | | */* | Unknown. |
| MUL_3364 | 80786.7 | 4.2693 | -0.313 | | */* | Unknown. |
| MUL_4441 | 37566.5 | 4.81 | -0.146 | | *phoS2* | Involved in active transport of inorganic phosphate across the membrane (import). This is one of the proteins required for binding-protein-mediated phosphate transport. |
| MUL_4697 | 16134.5 | 5.8679 | 0.254 | | *lprC* | Unknown. |
| MUL_4925 | 34399 | 4.6935 | -0.094 | | *drrA* | Daunorubicin-DIM-transport ATP-binding protein ABC |
| MUL_4926 | 25274.5 | 5.0508 | -0.095 | | */* | Unknown. |
| MUL_0714 | 33637.9 | 6.8137 | -0.557 | | */* | Unknown. |
| MUL_1602 | 55621.5 | 6.6249 | 0.463 | | *cydD* | Involved in active transport across the membrane of component linked with the assembly of cytochrome: involved in cytochrome biogenesis (aerobic respiration). responsible for energy coupling to the transport system and for the translocation of the substrate across the membrane. |
| MUL_2656 | 38561.8 | 6.1388 | -0.122 | | *phoS2_1* | Involved in active transport of inorganic phosphate across the membrane (import). this is one of the proteins. required for binding-protein-mediated phosphate transport. |
| MUL_3757 | 61815.8 | 4.913 | -0.392 | | */* | Thought to be involved in active transport of macrolides across the membrane (export): macrolide antibiotics resistance by an export mechanism. responsible for energy coupling to the transport system. |
| MUL_4543 | 22491.0 | 9.363 | -0.698 | | *hbhA* | mediates adherence to epithelial cells. |
| MUL_4567 | 37518.4 | 4.358 | -0.366 | | / | Unknown. |
| **7- Intermediary metabolism and respiration** | | | | | | |
| MUL_3557 | 40713.4 | 6.8567 | -0.109 | | *ctaC* | Involved in aerobic respiration. |
| MUL_0574 | 105322 | 6.9612 | -0.075 | | */* | Unknown. |
| MUL_1172 | 70530.6 | 5.8462 | -0.345 | | */* | Involved in interconversion of fumarate and succinate (aerobic respiration). |
| MUL_3092 | 152657.0 | 5.21 | -0.115 | | */* | Multiple FtsK/SpoIIIE protein domains. This domain contains a putative ATP binding p-loop motif and is found in the FtsK cell division protein from *E. coli* and the stage III sporulation protein E SpoIIIE. |
| MUL_3552 | 43085.3 | 6.5203 | -0.099 | | *qcrA* | Role in respiration. |
| MUL_3553 | 62187.4 | 7.6888 | 0.279 | | *qcrB* | Role in respiration. |
| MUL_3954 | 52686.5 | 4.559 | -0.156 | | *atpD* | ATP synthetase β chain. |
| MUL_4733 | 48455.7 | 5.8993 | 0.688 | | *pntB* | The transhydrogenation between NADH and NADP is coupled to respiration and ATP hydrolysis and functions as a proton pump across the membrane. |
| **10 –Conserved hypothetical Proteins** | | | | | | |
| MUL_3743 | 15876.8 | 4.6558 | 0.205 | | / | Unknown. |
| MUL_5022 | 23762.6 | 11.7999 | -0.404 | | / | Unknown. |
| MUL_2948 | 16657.8 | 10.3359 | -0.350 | | / | Unknown. |
| MUL_1499 | 16062.3 | 10.0934 | -0.294 | | / | Unknown . |
| MUL_4927 | 19195.3 | 10.4712 | -0.279 | | / | Unknown. |

Gray, Yellow and Green colors correspond to the proteins present in the three fractions ECM, membrane and vesicles, in both membrane and vesicles, in vesicles only respectively.
